# Elevated serum zonulin is associated with high attack frequency in hereditary angioedema: providing insight into the gut–angioedema axis

**DOI:** 10.1186/s13023-026-04286-6

**Published:** 2026-02-25

**Authors:** Ragıp Fatih Kural, Kasım Okan, Onurcan Yildirim, Elif Azarsiz, Serpil Akten, Reyhan Gumusburun, Ceyda Tunakan Dalgic, Nihal Mete Gokmen

**Affiliations:** 1 Division of Allergy and Immunology, Department of Internal Medicine, Ege University Faculty of Medicine, İzmir, Türkiye; 2 Department of Medical Biochemistry, Ege University Faculty of Medicine, İzmir, Türkiye

**Keywords:** Hereditary angioedema, Zonulin, Biomarker, Intestinal permeability, Psychological burden

## Abstract

**Background:**

Zonulin is a key regulator of epithelial barrier permeability and has been implicated in barrier dysfunction across various inflammatory conditions. To elucidate the potential role of intestinal permeability in HAE, this study investigated serum zonulin levels and evaluated their association with disease activity and psychiatric status.

**Patients and methods:**

The study included 28 patients with HAE type 1 (HAE-C1INH) and 30 age- and sex-matched healthy controls. Serum zonulin levels were measured by ELISA. Patients were stratified according to attack frequency (high: ≥2 vs. low: <2 attacks/month). Psychiatric status was assessed using the Hospital Anxiety and Depression Scale (HADS). Multivariable linear regression analysis was performed to evaluate the association between zonulin levels and attack frequency after adjustment for body mass index (BMI).

**Results:**

Overall serum zonulin levels did not differ significantly between patients with HAE and healthy controls (p = 0.06). However, stratified analyses revealed that patients with high attack frequency exhibited significantly higher zonulin levels compared with both low-attack-frequency patients and controls (p < 0.01), whereas zonulin levels in the low-attack-frequency group were comparable to controls. In multivariable analysis, attack frequency remained independently associated with serum zonulin levels after adjustment for BMI. Although zonulin levels were higher among patients with clinically significant anxiety and/or depressive symptoms based on HADS, this association was weaker than that observed for attack frequency.

**Conclusions:**

This pilot study provides preliminary evidence that elevated serum zonulin levels in HAE are associated with higher attack burden, rather than representing a uniform feature of the disease. These findings suggest that zonulin elevation reflects inflammatory activity independent of psychiatric symptom burden and position intestinal barrier integrity as a relevant mechanistic component within the proposed Gut–Angioedema Axis. Future prospective studies are warranted to validate these findings.

**Trial registration:**

Not applicable.

## Background

Hereditary angioedema (HAE) is a rare autosomal dominant disorder marked by recurrent episodes of angioedema affecting the extremities, face, gastrointestinal tract, and upper airways [1]. The disease primarily results from C1 inhibitor (C1INH) deficiency or dysfunction due to mutations in the *SERPING1* gene (HAE-C1INH Type 1 and Type 2) [2]. Loss of C1INH-mediated regulation disrupts the contact system, driving excessive conversion of prekallikrein to kallikrein and factor XII to activated factor XII. The resulting bradykinin surge activates bradykinin B2 receptors, triggering vasodilation and vascular leakage—the pathophysiological cornerstone of angioedema [3].

A subtype of HAE with normal C1INH (HAE-nC1INH) was recognized in the 2000s [4]; pathogenic variants in *F12*, *PLG*, *ANGPT1*, *KNG1*, *MYOF*, and *HS3ST6* have since been identified, with some affecting bradykinin production and others directly modulating vascular permeability [3, 5].

Clinical manifestations typically begin in childhood or adolescence and persist throughout life [6, 7]. Attacks may occur spontaneously [8] or follow identifiable triggers such as stress, trauma, infections, or hormonal fluctuations [6–9].

Beyond physical morbidity, HAE imposes considerable psychological burden. Anxiety affects 38–50% of patients and depression 14–24% of patients, largely attributable to attack unpredictability and fear of asphyxiation [10–12]. Attack frequency emerges as a critical determinant: patients with high attack frequency report markedly diminished health-related quality of life and work productivity relative to those with infrequent attacks [11, 13].

Zonulin, a regulator of intestinal tight junctions [14], modulates barrier permeability by disassembling intercellular protein complexes [15]. Zonulin upregulation induces intestinal barrier dysfunction (“leaky gut”), allowing translocation of luminal antigens into systemic circulation [16]. Elevated serum zonulin has been documented in gastrointestinal diseases (celiac disease [17], irritable bowel syndrome [18], inflammatory bowel disease [19], cirrhosis [20]), autoimmune conditions (type 1 diabetes [21], Hashimoto’s thyroiditis [22], multiple sclerosis [23]), allergic disorders (asthma [24], urticaria [25]), and neuropsychiatric disorders (anxiety/depression [26, 27], attention-deficit/hyperactivity disorder [28], schizophrenia [29]). Given that HAE pathophysiology centers on vascular permeability dysregulation and is associated with substantial psychiatric comorbidity, zonulin may function as a biomarker of barrier integrity and as a potential mediator linking disease activity with psychological burden in this population.

This study evaluates whether serum zonulin levels correlate with disease activity (specifically attack frequency) and psychiatric comorbidity in HAE patients. We further explore zonulin’s potential utility as a biomarker for identifying individuals at high risk for frequent attacks.

## Materials and methods

### Patients and controls

**Fig. 1:**
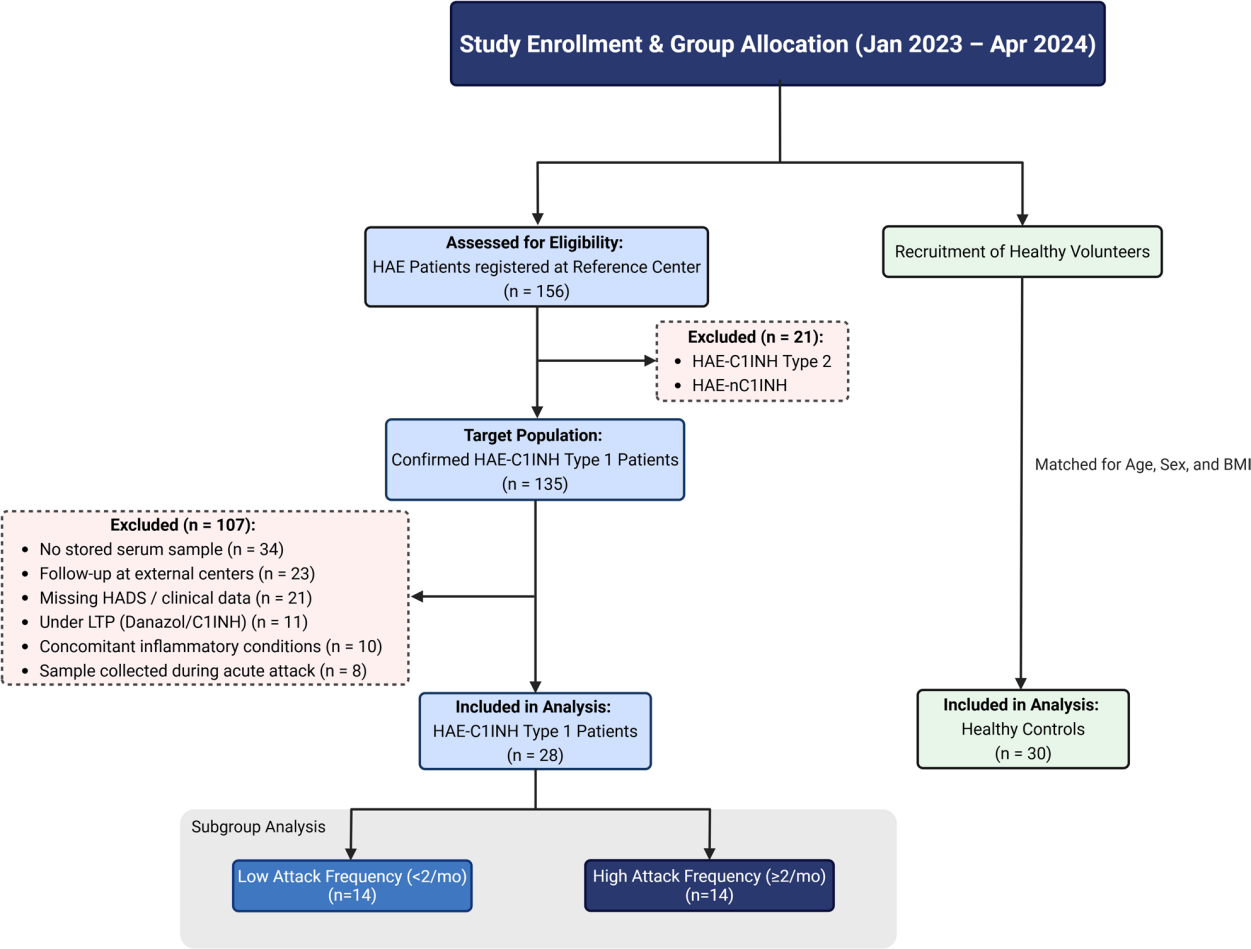
Flow diagram illustrating the study enrollment, eligibility assessment, and final group allocation of patients with HAE and healthy controls. Patients were identified from the institutional HAE registry and screened according to predefined inclusion and exclusion criteria. The final analysis included 28 patients with confirmed HAE type 1 (HAE-C1INH) and 30 age-, sex-, and body mass index–matched healthy controls. HAE patients were further stratified according to attack frequency into low (< 2 attacks/month) and high (≥ 2 attacks/month) attack frequency subgroups

This retrospective, observational, case-control study was conducted between January 2023 and April 2024 at the Department of Internal Medicine, Division of Adult Allergy and Immunology, Ege University Faculty of Medicine. The study population was selected from our institutional HAE registry (*n* = 156). The complete selection process, including all inclusion and exclusion criteria, is shown in the study flow diagram (Fig. 1). Following this screening, we included 28 patients with HAE-C1INH Type 1. A control group of 30 healthy individuals was matched for age, sex, and body mass index (BMI). The diagnosis of HAE-C1INH Type 1 was confirmed based on family history, clinical presentation, low serum C1INH levels, reduced C1INH functional activity, and, when necessary, genetic testing [2].

Individuals were excluded if they had any condition known to affect serum zonulin levels, such as inflammatory bowel disease, autoimmune disorders, malignancy, hematologic disease, use of systemic immunosuppressive therapy, chronic alcohol consumption, pregnancy, breastfeeding, or recent antibiotic use (within the past three months). These conditions were systematically screened through patient self-reports, medical records, and existing diagnostic documentation. The study was approved by the Ethics Committee of Ege University Faculty of Medicine, and written informed consent was obtained from all participants.

Data on age, sex, BMI, age at disease onset, disease duration, predominant attack site, and attack management were systematically collected from patients with HAE. The total number of attacks experienced by each patient in the 12 months prior to study inclusion was recorded and defined as the annual attack frequency. Disease activity was assessed using attack frequency. The predominant attack site was classified as either abdominal or non-abdominal (including extremities, face, or oropharyngeal region), and patients were divided into two groups accordingly. This classification was based on whether ≥ 50% of reported attacks occurred in the same anatomical region.

Information on acute attack treatments (e.g., icatibant), long-term prophylaxis (LTP), and previous therapies was collected and retrospectively reviewed using patient records and standardized forms. All patients with HAE were symptomatic and used on-demand icatibant for managing their attacks. Based on the annual attack frequency, patients were categorized into two groups: high attack frequency (≥ 2 attacks/month) and low attack frequency (< 2 attacks/month). Attack frequency was analyzed as a continuous variable using the annual number of attacks, while categorical subgrouping (high vs. low frequency) was defined using a monthly threshold (≥ 2 attacks/month).

### Psychiatric assessment

Psychiatric status in HAE patients was assessed using the Hospital Anxiety and Depression Scale (HADS), a validated screening tool composed of two subscales: anxiety (HADS-A) and depression (HADS-D). The scale consists of 14 items, with each subscale scored from 0 to 21. In our study, an abnormal HADS score was defined as a score of ≥ 11 on either the HADS-A or HADS-D subscale and was considered indicative of clinically significant anxiety or depressive symptom burden; this cutoff was also used to define subgroups for further analysis.

Data on current and past psychiatric disorders and the use of psychiatric medications were additionally collected from HAE patients. Current and past psychiatric disorders were identified from documented clinical diagnoses or ongoing psychiatric treatment in medical records, whereas HADS was used exclusively to assess symptom burden and not for diagnostic purposes.

### Measurement timing and sampling conditions

This study utilized archived serum samples that were collected and stored during standardized clinical follow-up visits. To ensure that zonulin levels and psychiatric assessments reflected the basal disease state, we verified each patient’s clinical status at the time of data collection. We confirmed that patients were attack-free for at least 72 h through a retrospective review of physical examination findings and patient-maintained diaries in the medical records. Furthermore, pharmacy records and clinical notes were cross-checked to ensure that all samples and HADS assessments were obtained prior to the initiation of LTP. This approach excluded the potential confounding effects of acute inflammation or prophylaxis on the study outcomes.

### Laboratory analysis of serum zonulin and complement parameters

Venous blood samples were collected from all participants after an overnight fast of at least 8 h. The samples were centrifuged at 3000 rpm for 10 min at room temperature, and the serum was separated and stored at − 80 °C until analysis. Serum zonulin levels were measured using a commercial Enzyme-Linked Immunosorbent Assay (ELISA) kit (Elabscience, Wuhan, China) in accordance with the manufacturer’s recommended protocols. All samples were analyzed in duplicate, and the intra-assay coefficient of variation (CV) remained below 10%.

To confirm the diagnosis of hereditary angioedema, serum C1INH level and function were assessed using standard laboratory methods and interpreted in accordance with reference ranges.

### Statistical analysis

Data were analyzed using SPSS version 25.0 (IBM Corp., Armonk, NY, USA). The distribution of variables was assessed using the Shapiro–Wilk test. Variables with a normal distribution are presented as mean ± standard deviation (SD), while non-normally distributed variables are expressed as median and interquartile range (IQR). Between-group comparisons were performed using the independent samples t-test or the Mann–Whitney U test, as appropriate. Categorical variables were analyzed using the chi-square test.

To explore the relationship between attack burden and intestinal permeability, patients were stratified according to attack frequency (high vs. low). Subgroup comparisons were performed to assess whether zonulin elevation represents a uniform feature across the disease spectrum or is predominantly associated with higher attack burden; accordingly, the high-attack-frequency and low-attack-frequency subgroups were independently compared with healthy controls using the Mann–Whitney U test.

Associations between serum zonulin levels and clinical parameters were assessed using Spearman correlation analysis. To evaluate whether the association between serum zonulin levels and attack burden was independent of BMI, a multivariable linear regression analysis was performed. Given the limited sample size and to preserve statistical power, a parsimonious model was constructed including only the number of attacks in the last year and BMI as independent variables, with serum zonulin level as the dependent variable. Regression coefficients (β), 95% confidence intervals (CI), and the coefficient of determination (*R²*) were reported. Model assumptions were checked using residual plots and normality assessments. In addition, zonulin levels were log-transformed and the multivariable analysis was repeated, yielding similar results. These analyses indicated that the observed association between attack frequency and zonulin levels was not dependent on data distribution.

Receiver operating characteristic (ROC) curve analysis was performed to assess the ability of serum zonulin levels to discriminate patients with high attack frequency. The optimal cutoff value was determined based on Youden’s index, and sensitivity, specificity, and area under the curve (AUC) values were calculated. A *p*-value < 0.05 was considered statistically significant.

Language editing was assisted by AI language tools; all content was reviewed and approved by the authors.

## Results

A total of 28 patients with HAE-C1INH Type 1 and 30 healthy controls were included in the study. As summarized in Table 1, the two groups were comparable in terms of age, sex distribution, and body mass index (*p* > 0.05). Serum zonulin levels were numerically higher in patients with HAE than in controls (median 74.5 vs. 44.9 ng/mL); however, this difference did not reach statistical significance (*p* = 0.06).

Among patients, the mean disease duration was 34.3 ± 16.7 years, and symptom onset was observed at an early age (median: 7.5 [4.8–12.2] years). The median number of attacks reported in the last year was 23.0 [15.75–49.0].

Psychiatric status evaluation revealed that 21.4% of patients had a current psychiatric disorder based on documented clinical diagnoses, including anxiety disorders (14.3%) and depressive disorders (7.1%). Additionally, 32.1% of patients had a history of psychiatric disorders for which they had received medical treatment. Furthermore, 12 patients (42.9%) had an abnormal HADS score, indicating a higher psychiatric symptom burden related to anxiety and/or depression. Detailed data are presented in Table 1.

**Table 1:** Demographic and clinical characteristics of the study population

Characteristic	HAE patients	Controls	*p*-value
**Demographics and Biomarkers**			
Gender, n (%)			
Male Female	10 (35.7) 18 (64.3)	13 (43.3) 17 (56.7)	0.74
Age, years (mean ± SD)	44.4 ± 18.5	43.2 ± 12.6	0.77
BMI, kg/m² (mean ± SD)	26.4 ± 4.1	26.0 ± 4.5	0.45
Serum Zonulin Level, (ng/mL)	74.5 (38.3–138.7)	44.9 (33.0–98.3)	0.06
**HAE Disease Characteristics**			
Disease Onset Age	7.5 (4.8–12.2)	-	-
Disease Duration, (yr)	34.3 ± 16.7	-	-
Dominant Attack Localization, n (%)			
Abdominal Non-abdominal	14 (50.0) 14 (50.0)	-	-
Annual Attack Frequency, n	23.0 (15.75–49.0)	-	-
**Psychiatric Status**			
Current Psychiatric Diagnosis, n (%)	6 (21.4)	-	-
History of Psychiatric Disorder, n (%)	9 (32.1)	-	-
HADS Total Score (mean ± SD)	15.9 ± 9.4	-	-
Abnormal HADS Score, n (%)	12 (42.8)	-	-

Values are presented as mean ± standard deviation (SD), median (interquartile range [IQR] or min-max), or number (percentage). BMI: Body Mass Index; HADS: Hospital Anxiety and Depression Scale. -: Not applicable. Annual attack frequency represents the number of attacks in the last year

Serum zonulin levels were significantly higher in the high attack frequency group compared to the low attack frequency group (median: 147.4 [99.5–236.7] ng/mL vs. 37.5 [31.6–46.9] ng/mL; *p* < 0.01). When these subgroups were independently compared with the healthy control group, serum zonulin levels in the high-attack-frequency group were found to be significantly higher than those in healthy controls (*p* < 0.01). In contrast, no statistically significant difference was observed between the low-attack-frequency group and healthy controls (*p* = 0.38).

BMI was also significantly higher in the high attack frequency group compared to the low attack frequency group (27.7 ± 4.3 kg/m² vs. 23.7 ± 2.1 kg/m²; *p* < 0.01). Although current psychiatric disorders (35.7% vs. 7.1%, *p* = 0.16) and a history of psychiatric disorders (50.0% vs. 14.3%, *p* = 0.10) were more common in the high attack frequency group, these differences did not reach statistical significance. Other HADS-related parameters (HADS total, HADS-A, HADS-D scores, and HADS-A or HADS-D ≥ 11) were also higher in the high attack frequency group, but these differences were not statistically significant (*p* > 0.05). Regarding treatment characteristics, 7 patients in the high-attack-frequency group were receiving LTP, including plasma-derived C1-inhibitor (*n* = 3) and danazol (*n* = 4), whereas none of the patients in the low-attack-frequency group were on LTP at the time of sampling. Detailed data are presented in Table 2.

**Table 2:** Demographic and clinical characteristics of HAE patients stratified by attack frequency

Characteristic	Low Frequency (< 2 attacks/mo) (*n* = 14)	High Frequency (≥ 2 attacks/mo) (*n* = 14)	*p*-value
**Demographics and Biomarkers**			
Gender n, (%)			0.26
Male Female	7 (50%) 7 (50%)	3 (21.4%) 11 (78.6%)	
Age, years (mean ± SD)	42.4 ± 19.5	46.5 ± 18	0.44
BMI, kg/m² (mean ± SD)	23.7 ± 2.1	27.7 ± 4.3	**< 0.01**
Serum Zonulin Level, (ng/mL)	37.5 (31.6–46.9)	147.4 (99.5–236.7)	**< 0.01**
**HAE Disease Characteristics**			
Disease Onset Age, years	8.5 (5.0–13.5)	7.0 (4.5–11.5)	0.85
Disease Duration, years	31.07 ± 16.99	37.57 ± 16.29	0.34
Annual Attack Frequency, n	15.5 (7.0–18.0)	50.0 (37.8–56.0)	**< 0.01**
LTP, n (%)	0 (0)	7 (50.0)	**< 0.01**
**Psychiatric Status**			
Current Psychiatric Diagnosis, n (%)	1 (7.1%)	5 (35.7%)	0.16
History of Psychiatric Disorder, n (%)	2 (14.3%)	7 (50.0%)	0.10
HADS Total Score (mean ± SD)	13.79 ± 6.97	18.07 ± 11.32	0.24
Abnormal HADS Score, n (%)	3 (21.4%)	9 (64.3%)	0.06

Values are presented as mean ± standard deviation (SD), median (interquartile range [IQR] or min-max), or number (percentage). BMI: Body Mass Index; HADS: Hospital Anxiety and Depression Scale; LTP: Long-term prophylaxis. Bold values indicate statistical significance (*p* < 0.05). Annual attack frequency represents the number of attacks in the last year. Note: LTP status indicates treatment initiated after sample collection; all serum samples were obtained prior to LTP initiation

Patients were grouped based on their psychiatric status, and the results of comparative analyses of serum zonulin levels are presented in Table 3. Serum zonulin levels were significantly higher in patients with a current psychiatric disorder (*p* < 0.01) and in those with abnormal HADS scores (*p* = 0.04). Zonulin levels were also higher in patients with a history of psychiatric disorders; however, this difference did not reach statistical significance (*p* = 0.081).

**Table 3:** Comparison of serum zonulin levels based on HADS scores and psychiatric status in patients with hereditary angioedema

Comparison Groups	*n*	Median Zonulin [Median (IQR)]	*p*-value
**HADS Score Classification**			
Normal / Borderline (< 11) Abnormal (≥ 11)	16 12	44.17 (35.31–96.99) 115.85 (65.71–210.60)	**0.04**
**Current Psychiatric Diagnosis**			
Absent Present	22 6	52.58 (35.67–98.20) 181.22 (138.72–236.74)	**< 0.01**
**History of Psychiatric Disorder**			
Absent Present	19 9	56.82 (35.72–97.59) 130.00 (78.90–197.54)	0.08

Values are presented as median (interquartile range [IQR]). HADS: Hospital Anxiety and Depression Scale. Bold values indicate statistical significance (*p* < 0.05)

Patients were also divided into two groups based on the predominant attack localization: abdominal (*n* = 14) and non-abdominal (*n* = 14). Although the median serum zonulin level was higher in the abdominal-dominant group, the difference was not statistically significant (*p* = 0.55).

According to Spearman correlation analysis, serum zonulin levels were significantly positively correlated with the number of attacks in the last year (*r* = 0.82, *p* < 0.01) and with BMI (*r* = 0.59, *p* < 0.01). No significant correlations were found between serum zonulin levels and other parameters, including C1INH level, C1INH function, disease duration, age, sex, total HADS score, or HADS-A and HADS-D subscores (Table 4).

**Table 4:** Spearman correlation analysis of zonulin levels and clinical parameters in hereditary angioedema

Variables	Spearman’s *r*	*p*-value
Age	-0.00	0.99
BMI	0.59	**< 0.01**
C1INH Level	0.25	0.20
C1INH Function	0.24	0.21
Disease Duration	0.09	0.66
Annual Attack Frequency	0.82	**< 0.01**
HADS Total Score	0.198	0.31
HADS-A Score	-0.045	0.82
HADS-D Score	0.315	0.10

BMI: Body Mass Index; C1INH: C1 inhibitor; HADS: Hospital Anxiety and Depression Scale (HADS-A: Anxiety subscale, HADS-D: Depression subscale). Bold values indicate statistical significance (*p* < 0.05)

**Fig. 2:**
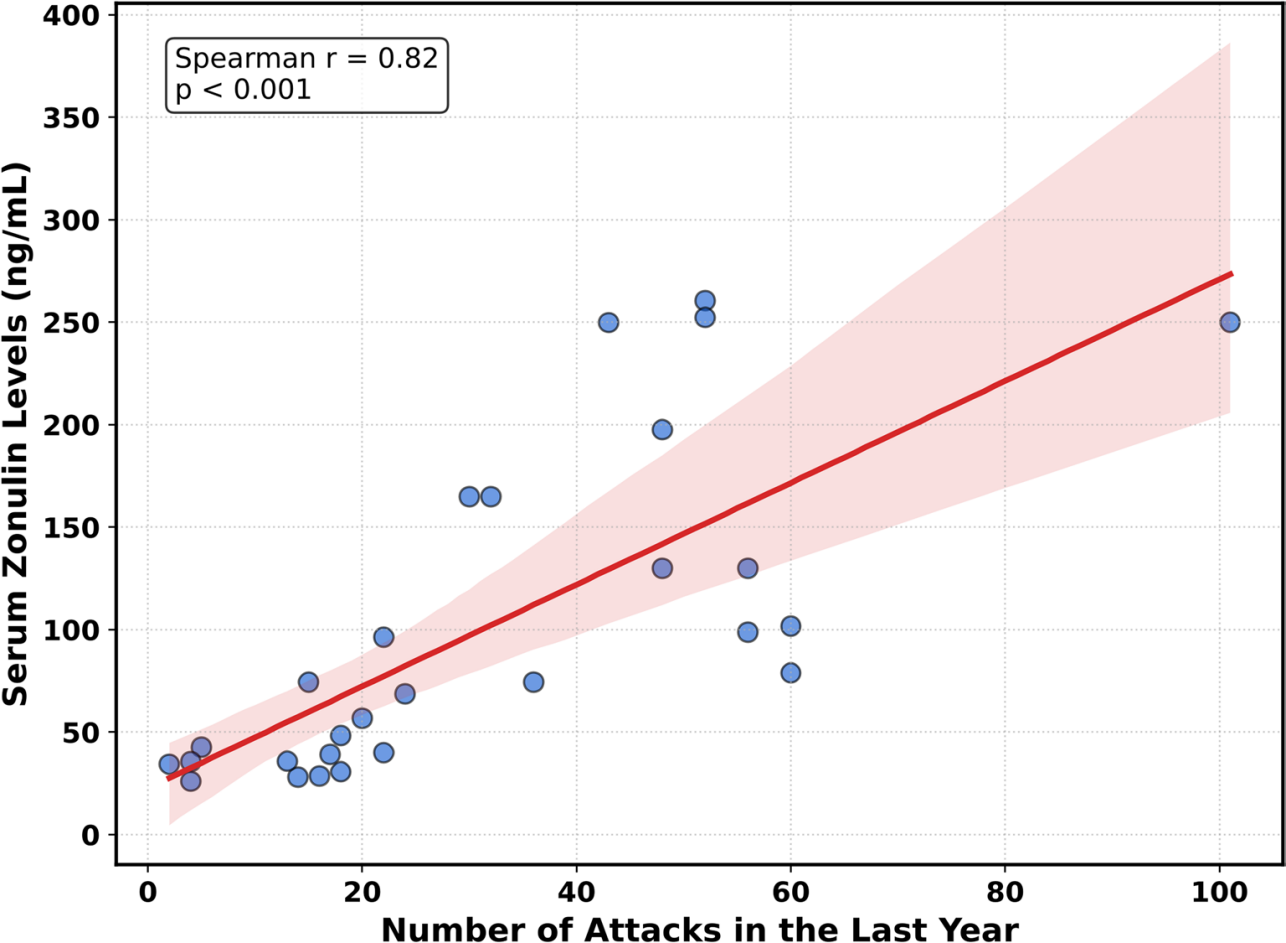
Correlation between annual attack frequency and serum zonulin levels. Scatter plot and linear regression analysis demonstrating the relationship in patients with HAE. The graph displays the number of attacks recorded in the last year plotted against serum zonulin concentrations

Linear regression analysis showed that each additional attack in the last year was associated with an average increase of 2.48 ng/mL in serum zonulin levels (*R²* = 0.54, *p* < 0.01) (Fig. 2). To assess whether this association was influenced by body mass index, we performed a multivariable linear regression analysis. In this adjusted model, the number of attacks in the last year remained a significant independent predictor of serum zonulin levels (β = 2.12, 95% CI: 0.99–3.24; *p* = 0.001), whereas BMI was not independently associated with zonulin levels (*p* = 0.25) (Table 5).

**Table 5:** Multivariable linear regression analysis evaluating factors associated with serum zonulin levels

Predictor	β coefficient	95% CI	*p*-value
Annual Attack Frequency	2.12	0.99–3.24	**0.001**
Body mass index (kg/m²)	3.76	-2.84–10.36	0.25

Dependent variable: serum zonulin levels. β, unstandardized regression coefficient; CI, confidence interval. Adjusted R² = 0.53. Annual attack frequency represents the number of attacks in the last year. Bold values indicate statistical significance (*p* < 0.05)

**Fig. 3:**
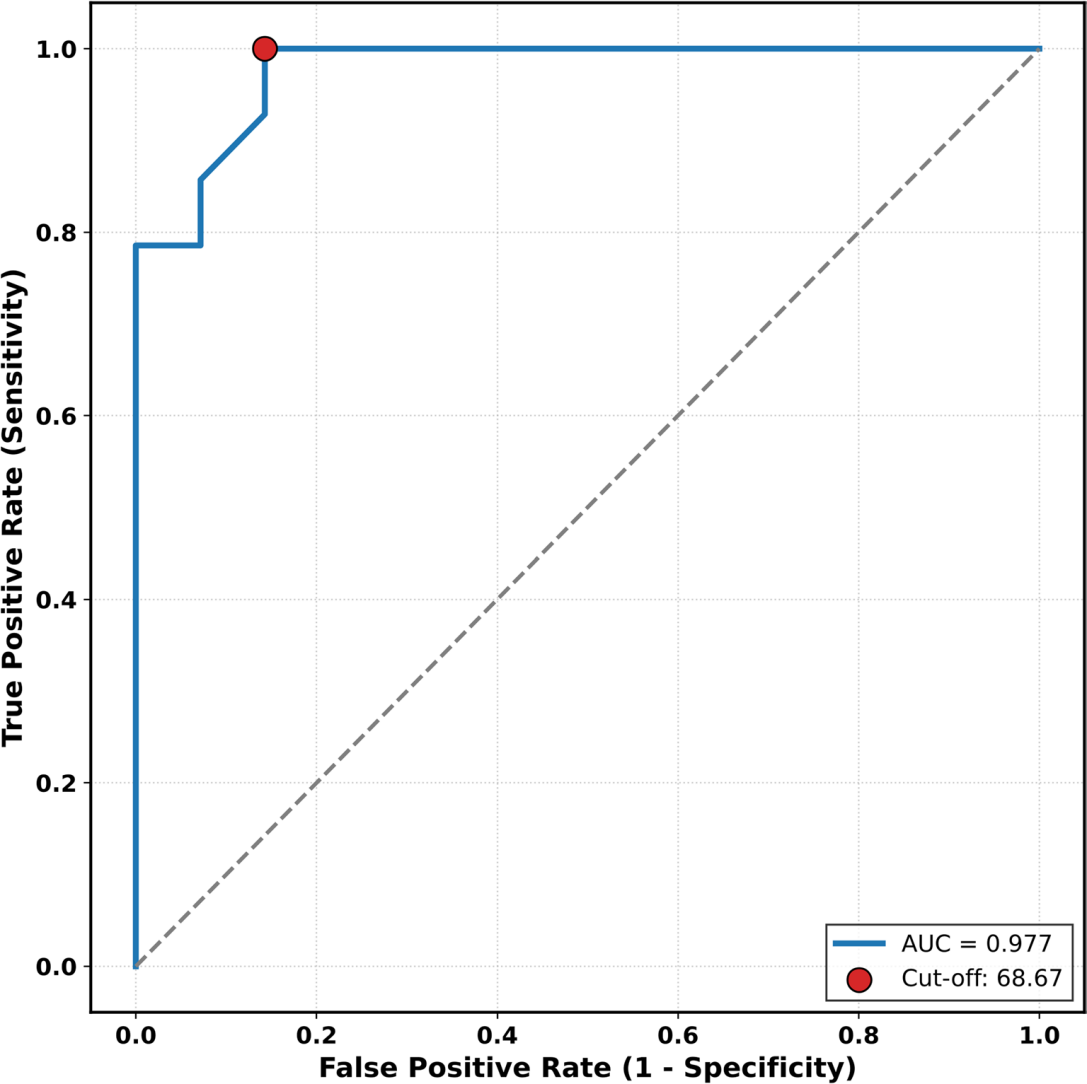
ROC curve analysis of serum zonulin levels. The curve illustrates the performance of serum zonulin in identifying HAE patients with high attack frequency (≥ 2 attacks/month). The area under the curve (AUC) is 0.977, indicating high discriminative ability

ROC curve analysis demonstrated a high discriminative performance of serum zonulin levels for identifying patients with high attack frequency, with an area under the curve (AUC) of 0.977 (*p* < 0.001). A zonulin cutoff value of 68.67 ng/mL yielded a sensitivity of 100% and a specificity of 85.7% for high attack frequency in this cohort (Fig. 3).

## Discussion

In this study, we examined serum zonulin levels in patients with HAE and explored their associations with attack burden and psychiatric symptom burden. Zonulin levels were numerically higher in the HAE group than in healthy controls, but the overall between-group difference was not statistically significant. Importantly, stratification by attack frequency showed that patients with a high attack frequency (≥ 2 attacks/month) had higher zonulin levels, whereas patients with a low attack frequency did not differ from controls. These findings suggest that elevated zonulin is not a uniform feature of HAE, but may be associated with higher attack burden within the HAE cohort.

Zonulin is an endogenous regulator of tight junctions involved in the modulation of intestinal epithelial permeability [14, 30]. Increased zonulin activity is associated with disruption of intercellular junctions and enhanced translocation of gut-derived antigens into the systemic circulation. This “leaky gut” phenomenon is increasingly recognized as a potential contributor to systemic inflammation and has been implicated in neuroinflammatory pathways linked to psychiatric distress [16]. Given that HAE is fundamentally a disorder of vascular permeability, exploring whether a parallel impairment exists at the level of the intestinal barrier provides a biologically plausible mechanistic hypothesis.

Recent reports suggest that gut and throat microbiota composition in HAE may vary in relation to disease activity and attack localization [31, 32]. In patients who recently experienced an attack, decreased gut microbial diversity, a reduction in Firmicutes species, and an increase in Proteobacteria have been reported—patterns that have been linked to impaired intestinal epithelial barrier integrity. Moreover, increased abundance of potentially pathogenic species such as *Klebsiella* and *Morganella* has been associated with greater disease severity [32]. In addition, alterations in upper airway microbiota have been described in patients with laryngeal edema [31]. Prior work has also proposed *Helicobacter pylori* infection as a potential trigger in some patients, with reduced attack frequency observed following eradication therapy [33].

**Fig. 4:**
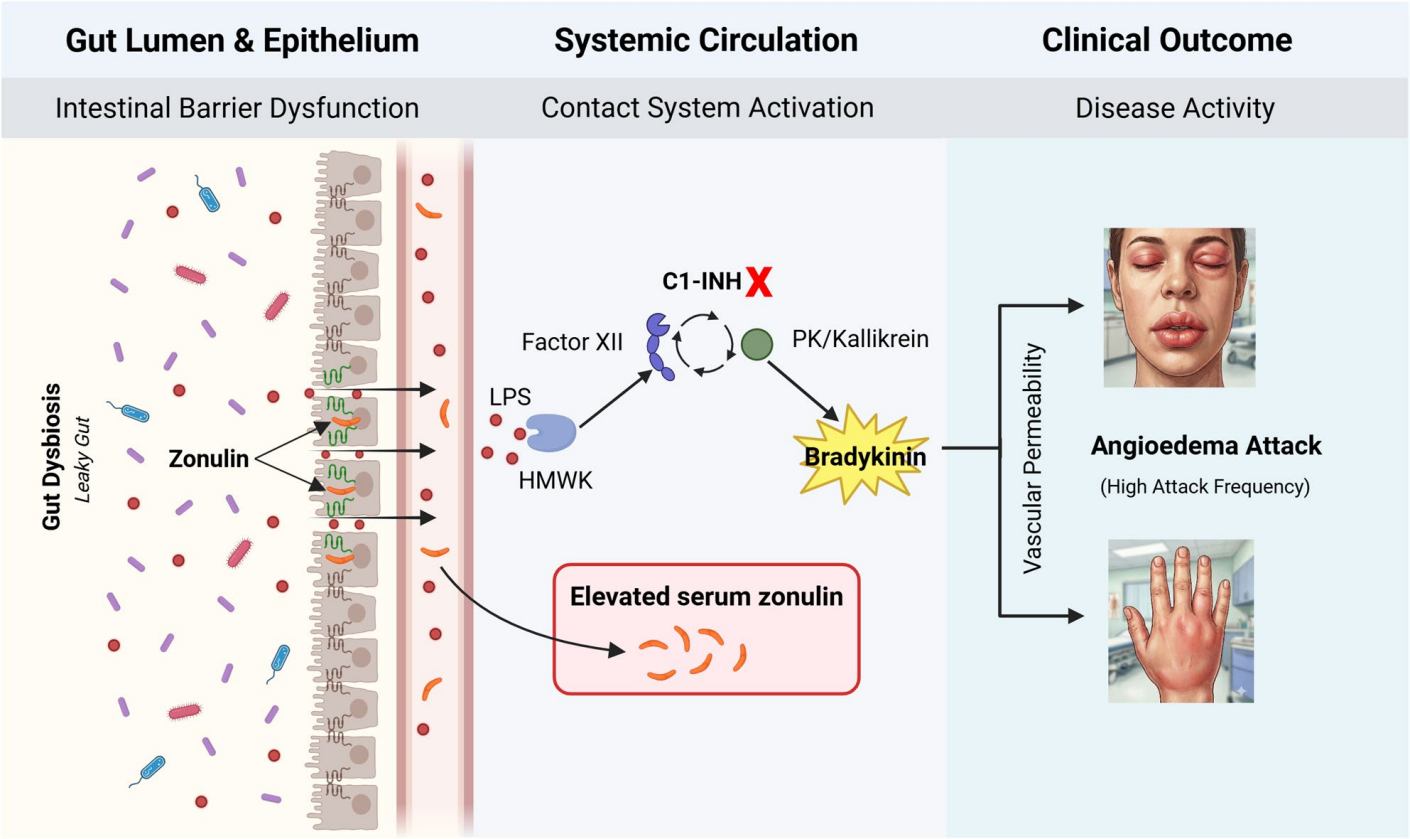
Proposed “Gut–Angioedema Axis” in HAE. Increased serum zonulin levels indicate impaired intestinal barrier integrity (**Left Panel**), facilitating the paracellular translocation of gut-derived microbial products (e.g., LPS) into the systemic circulation. In the bloodstream (**Middle Panel**), the LPS-HMWK complex may lower the threshold for contact system activation. In the presence of C1-Inhibitor deficiency, this trigger leads to uncontrolled kallikrein activity and excessive bradykinin production. Consequently, this cascade promotes vascular permeability and angioedema attacks (**Right Panel**). (Figure created with BioRender.com). Abbreviations: HAE, hereditary angioedema; LPS, lipopolysaccharide; HMWK, high-molecular-weight kininogen; PK, prekallikrein; C1-INH, C1 inhibitor

Collectively, these findings suggest that microbiota alterations may influence HAE attacks not only through local effects but also by promoting systemic inflammatory responses and increasing vascular permeability. Indeed, various microorganisms can directly or indirectly activate the contact and kallikrein–kinin systems, leading to increased bradykinin production [34]. In particular, microbial products such as lipopolysaccharide (LPS) can interact with high-molecular-weight kininogen and activate the contact system [35]. This mechanism provides a biologically plausible link between intestinal barrier dysfunction, systemic inflammation, and attack initiation in HAE (Fig. 4).

A relationship between intestinal permeability and psychological distress has previously been suggested. For example, Stevens et al. (2018) reported significantly higher plasma zonulin and LPS levels in individuals with depression or anxiety [27]. Given that HAE is characterized by a substantial neuropsychiatric burden—as documented in multinational cohorts [10–12] and in our previous experience in Türkiye [36]—we hypothesized that a potential “Gut–Angioedema Axis” interaction might exist in HAE. Accordingly, we sought to clarify whether serum zonulin levels in HAE are driven primarily by psychiatric symptom burden or by the intrinsic inflammatory activity of the disease.

Within our HAE cohort, a considerable proportion of patients had a current psychiatric diagnosis or clinically significant anxiety and/or depression based on HADS scores. When patients were grouped according to psychiatric status, serum zonulin levels were higher in those with a current psychiatric disorder. However, an important concern regarding zonulin is its potential lack of specificity due to its association with general psychological distress [26–29]. Notably, in our study, serum zonulin levels showed a significant association with attack frequency, whereas correlations with HADS scores were not statistically significant. This pattern suggests that zonulin elevation in HAE may be more closely associated with attack burden than with psychological distress alone. This interpretation is further supported by the observation that zonulin levels were elevated exclusively in the high-attack-frequency group, while no difference was observed in patients with low attack frequency. Together, these findings indicate that zonulin elevation is not a uniform feature of HAE but is preferentially associated with higher attack burden rather than psychiatric comorbidities.

Patients in the high-attack-frequency subgroup also exhibited higher BMI values, and a positive correlation was observed between serum zonulin levels and BMI. This raises the possibility that elevated zonulin levels could reflect metabolic status, as suggested in previous obesity-related studies [16]. However, multivariable linear regression analysis demonstrated that attack frequency remained independently associated with serum zonulin levels after adjustment for BMI, whereas BMI itself was not independently associated with zonulin levels. These findings support the interpretation that zonulin elevation in this cohort is primarily related to disease activity rather than metabolic confounding.

Consistent with these observations, regression analysis indicated that each additional attack per year was associated with an increase in serum zonulin levels. In exploratory analyses, a serum zonulin cutoff value of 68.67 ng/mL was identified for distinguishing patients with high attack frequency. This finding should be interpreted strictly as hypothesis-generating, as the analysis was exploratory, derived from a single cohort, and lacks external validation.

## Conclusions

To our knowledge, this is the first study to evaluate serum zonulin levels in patients with HAE. Our findings provide preliminary evidence that elevated zonulin is associated with higher attack burden rather than representing a uniform feature of the disease and appears to be independent of psychiatric comorbidities. Although the clinical applicability of zonulin measurements requires further validation, these results position intestinal barrier integrity as a key mechanistic component within the proposed “Gut–Angioedema Axis.” In this context, favorable results observed with barrier-targeted agents such as larazotide acetate in other inflammatory disorders [37] support the translational relevance of this pathway. Future prospective studies incorporating microbiota analyses and longitudinal assessments are warranted to clarify whether targeting intestinal permeability could offer novel therapeutic strategies for HAE.

### Limitations

This study has several limitations that should be acknowledged. First, although focusing exclusively on HAE-C1INH Type 1 ensured sample homogeneity, this design choice limits the generalizability of our findings to other HAE subtypes, including HAE-C1INH Type 2 and HAE-nC1INH. Second, intestinal permeability was assessed solely using serum zonulin; future studies incorporating a broader biomarker panel—including LPS, intestinal fatty acid–binding protein, and claudin-3—alongside microbiota analyses would provide a more comprehensive evaluation of barrier integrity. Third, because measurements were restricted to attack-free periods, zonulin dynamics during acute HAE attacks remain unexplored, highlighting the need for prospective time-series studies.

Additionally, the retrospective design limited disease severity assessment to attack frequency rather than validated disease activity scores such as the Angioedema Activity Score, thereby constraining precise characterization of disease burden. The absence of systematic psychiatric data for the control group precluded direct comparison of zonulin–psychiatric symptom relationships between patients and controls. Finally, ROC analyses were exploratory in nature and require validation in larger, prospective cohorts.

## Data Availability

The datasets generated and analyzed during the current study are available from the corresponding author on reasonable request.

## Abbreviations

AUC: Area Under the Curve
BMI: Body Mass Index
C1INH: C1 Inhibitor
CV: Coefficient of Variation
ELISA: Enzyme-Linked Immunosorbent Assay
HAE: Hereditary Angioedema
HAE-C1INH: Hereditary Angioedema due to C1 Inhibitor Deficiency
HAE-nC1INH: Hereditary Angioedema with Normal C1 Inhibitor
HADS: Hospital Anxiety and Depression Scale
HADS-A: HADS – Anxiety Subscale
HADS-D: HADS – Depression Subscale
IQR: Interquartile Range
LPS: Lipopolysaccharide
LTP: Long-Term Prophylaxis
ROC: Receiver Operating Characteristic
SD: Standard Deviation
